# Using Data Linkage to Investigate Inconsistent Reporting of Self-Harm and Questionnaire Non-Response

**DOI:** 10.1080/13811118.2015.1033121

**Published:** 2016-01-20

**Authors:** Becky Mars, Rosie Cornish, Jon Heron, Andy Boyd, Catherine Crane, Keith Hawton, Glyn Lewis, Kate Tilling, John Macleod, David Gunnell

**Keywords:** agreement, ALSPAC, consistency, data linkage, self-harm, suicide attempt

## Abstract

The objective of this study was to examine agreement between self-reported and medically recorded self-harm, and investigate whether the prevalence of self-harm differs in questionnaire responders vs. non-responders. A total of 4,810 participants from the Avon Longitudinal Study of Parents and Children (ALSPAC) completed a self-harm questionnaire at age 16 years. Data from consenting participants were linked to medical records (number available for analyses ranges from 205–3,027). The prevalence of self-harm leading to hospital admission was somewhat higher in questionnaire non-responders than responders (2.0 vs. 1.2%). Hospital attendance with self-harm was under-reported on the questionnaire. One third reported self-harm inconsistently over time; inconsistent reporters were less likely to have depression and fewer had self-harmed with suicidal intent. Self-harm prevalence estimates derived from self-report may be underestimated; more accurate figures may come from combining data from multiple sources.

## INTRODUCTION

Community studies of self-harm are vital as the majority of self-harm episodes do not present to clinical services (Hawton, Rodham, Evans, & Weatherall, 2002; Kidger, Heron, Lewis, Evans, & Gunnell, 2012; Ystgaard et al., 2009). However, such studies are subject to a number of limitations such as misreporting and non-response (Grimes & Schulz, 2002), which can lead to bias in estimates of prevalence and measures of association. The extent to which this occurs in the case of self-harm is not currently known.

Non-response and loss to follow-up occur more frequently among individuals with particular characteristics (Kidger et al., 2012; Wolke et al., 2009). For example, in the Early Developmental Stages of Psychopathology Study (Christl, Wittchen, Pfister, Lieb, & Bronisch, 2006), participation rates at follow-up were lower among those who had attempted suicide compared to those without suicidal thoughts or attempts. This skewed pattern of participation would have led to underestimates of the prevalence of suicide attempts.

In addition, as information on self-harm is typically collected retrospectively via self-report, the accuracy of responses may be affected by issues such as denial, reinterpretation, problems with recall, current mood, or by misinterpretation of the study questions (Velting, Rathus, & Asnis, 1998). There is also evidence to suggest that concerns over social desirability may encourage under-reporting, as adolescents have been found to report suicide attempts two to three times more frequently under conditions of anonymity (Safer, 1997).

The agreement between different sources of data on self-reported self-harm in adolescents has previously been investigated (Bjärehed, Pettersson, Wångby-Lundh, & Lundh, 2013; O'Sullivan & Fitzgerald, 1998; Ougrin & Boege, 2013; Ross & Heath, 2002; Velting et al., 1998). Most have focused on suicide attempts, which comprise only a portion of episodes (Hawton et al., 2002; Kidger et al., 2012; Muehlenkamp & Gutierrez, 2004). Studies have reported inconsistency across different self-report methods, e.g., interviews vs. questionnaires (Bjärehed et al., 2013; O'Sullivan & Fitzgerald, 1998; Ougrin & Boege, 2013; Ross & Heath, 2002; Velting et al., 1998), and also when using the same self-report method across repeated assessments (Christl et al., 2006; Hart, Musci, Ialongo, Ballard, & Wilcox, 2013). However, the absence of a gold standard means it is not possible to tell which measure/assessment is more accurate (see Table 1 for a summary of studies). There is some evidence to suggest that individuals with more severe psychopathology are more likely to report self-harm consistently over time (Christl et al., 2006; Eikelenboom, Smit, Beekman, Kerkhof, & Penninx, 2014).

**TABLE 1.** Studies Investigating Consistency in Self-Harm Reports: Across Different Measures or Over Time

Publication	Country	Sample	Measure	Comparison of different measures	Consistency in reports over time	Other design	Results
Adolescent samples							
Ougrin and Boege (2013)	United Kingdom	Adolescent inpatients and outpatients *n* = 100, 12–17 years	Self-harm	Yes Self-report questionnaire and clinical record (reported during the clinical assessment)	No	No	3 (3%) indicated in the clinical record they had self-harmed but did not report self-harm on the questionnaire. 20 (20%) reported at least one episode of self-harm on the questionnaire that was not recorded in the clinical record
Hart et al. (2013)	United States	Longitudinal community sample of adolescents *n* = 678, Assessed annually between age 12 and 22 years	Suicide attempts	No	Yes Also examined characteristics associated with discrepant reporting	No	88.5% inconsistently reported a suicide attempt at some point during the study; 65.3% were inconsistent the year following the self-harm event Consistent and inconsistent reporters did not differ on clinical or demographic variables, but consistent reporters had higher lifetime suicidal ideation
Bjärehed et al. (2013)	Sweden	Adolescent community sample *n* = 1,052, from grade 7 (mean age 13.7 years) and 8 (mean age 14.7 years)	Non-suicidal self-injury	Yes Self-report questionnaire and follow-up interview	No	No	97 adolescents were selected for interview. 32/66 (48%) participants who reported self-harm on the questionnaire did not disclose self-harm during the follow-up interview.
Kidger et al. (2012)	United Kingdom	Adolescent community sample *n* = 4810, 16 years	Suicide attempts	No	No	Yes Examined suicidal thoughts among those with suicidal self-harm	Approximately 10% of those who reported wanting to die during the most recent episode of self-harm said they had never had thoughts of killing themselves
Christl et al. (2006)	Germany	Longitudinal community sample of adolescents/young adults *n* = 3021, 14–24 years at baseline	Suicide attempts	No	Yes Also examined characteristics associated with discrepant reporting	Yes Compared drop-out rates among those with and without suicidal behaviour	One third of baseline suicide attempters (*n* = 15/45), did not report a suicide attempt at follow-up 4 years later 81% of discrepant reporters were female and 59% were aged 14–17 at baseline. Greater consistency in reporting was associated with a higher number of psychiatric disorders Those with a suicide attempt at baseline were at least 1.6 times more likely to drop out of the study then those without suicide attempts or ideas
Ross and Heath (2002)	Not specified	Adolescent community sample *n* = 440 (from 2 schools), average age 14–15 years	Self-mutilation (defined as deliberate alteration or destruction of body tissue without suicidal intent)	Yes Self-report screening questionnaire and follow-up interview	No	No	School sample 1: 38.8% who reported self-harm on the questionnaire were not classified as having self-harmed following the interview (19/49) School sample 2: 24.4% who reported self-harm on the questionnaire were not classified as having self-harmed following the interview (10/41)
Velting et al. (1998)	United States	Adolescent outpatients *n* = 48, 12–20 years, mean 15.3 years	Suicide attempts	Yes Self-report questionnaire and interview Also investigated explanations and characteristics associated with discrepant reporting	No	No	Discrepancies in reporting were found amongst 50% of the sample (24/48). Discrepancies primarily due to confusion with the operational definition of suicidal behaviour (i.e., confused attempt and ideation or confused attempt and gesture). The discrepant and non-discrepant groups were comparable on measures of suicidal intent, ideation, and hopelessness and on their diagnostic profiles
O'Sullivan and Fitzgerald (1998)	Ireland	Adolescent community sample *n* = 88 age 13–14 years	Suicide attempt	Yes Self-report screening questionnaire and follow-up interview	No	No	45 adolescents completed a follow-up interview. 5/7 (71%) participants who reported a suicide attempt on the questionnaire did not disclose self-harm during the follow-up interview.
Adult samples							
Eikelenboom et al. (2014)	The Netherlands	Longitudinal cohort of adults with depressive or anxiety disorders *n* = 1973, aged 18–65 years at baseline, (mean age 42.4 years)	Suicide attempts	No	Yes Also examined characteristics associated with discrepant reporting	No	23% of baseline suicide attempters, did not report their attempt at follow-up 2 years later (63/274) Consistent reporting was associated with a greater number of suicide attempts, and more severe current psychopathology. No differences were found for recency of the event, age, sex, or education
Morthorst et al. (2011)	Denmark	Patients admitted to hospital following a suicide attempt *n* = 243, age 12+, mean age 31 years	Suicide attempts, assessed 1 year after baseline	Yes Self-report (telephone interview) and hospital records	No	No	Seven suicide attempts listed in the hospital records were not reported by participants. Nine patients reported a suicide attempt that was not listed in the hospital records
Plöderl et al. (2011)	Austria	Adult community sample *n* = 1385, age 18–84 years, Mean 37.8 years	Suicide attempts	No	No	Yes Examined intent to die among those reporting suicidal self-harm	One quarter (15/60) of individuals reporting a suicide attempt were false positives (lacked intent or attempt aborted) 0.8% (*n* = 11) were identified as false negatives (reported no suicide attempt on the screen question but reported a self-harm event with intent to die in follow-up questions). 2/11 (18%) false negatives resulted in injuries requiring hospital treatment There were no differences between true positives and false positives regarding age or education or lethality of method
Linehan et al. (2006)	United States	Adult clinical sample: Five cohorts, three with borderline personality disorder	Self-harm	Yes Self-report interview and 1) therapist notes 2) participant diary cards 3) medical records	No	No	Agreement with therapist notes (presence/absence of self-harm) was 83% Good agreement with diary cards (mean 4.5 acts at interview vs. mean 4.3 acts on diary cards) 82% of episodes reported by participants as being medically treated had a corresponding medical record. There were no false negatives —all medically treated episodes were reported by participants.
Nock and Kessler (2006)	United States	Predominately Adult community sample *n* = 5,877, aged 15–54 years	Suicide attempts	No	No	Yes Examined intent to die among those reporting suicidal self-harm	112/268 (42%) of those reporting a lifetime history of suicide attempt reported no intent to die

Whereas previous studies have typically compared self-report questionnaire and interview responses, the present study compares self-reported self-harm with data from medical records. This data is external and objective, although it cannot be considered to be free from error. We linked data from medical records with data reported by participants in the Avon Longitudinal Study of Parents and Children, a longitudinal population-based birth cohort (Boyd et al., 2013). Our aims were to:

1. Investigate whether the prevalence of self-harm recorded in medical records differs between responders and non-responders to the self-harm questionnaire.
2. Investigate the level of agreement between self-report and medically recorded self-harm events.
3. Examine consistency in the reporting of self-harm in ALSPAC over time, by comparing questionnaire responses at age 16 and 18 years.
4. Identify characteristics associated with inconsistent reporting of self-harm over time.

We hypothesize that the prevalence of self-harm recorded in medical records will be higher among questionnaire non-responders than responders, and that recall of self-harm episodes over time will be most consistent in individuals with more severe mental health problems/self-harm.

## METHODS

### Sample

#### The Avon Longitudinal Study of Parents and Children (ALSPAC)

ALSPAC is a population-based birth cohort study examining influences on health and development across the lifecourse. The ALSPAC core enrolled sample consists of 14,541 pregnant women resident in the former county of Avon in South West England (United Kingdom), with expected delivery dates between April 1, 1991 and December 31, 1992 (Boyd et al., 2013). Of the 14,062 live births, 13,798 were singletons/first-born of twins and were alive at 1 year of age. Participants have been followed up since recruitment through regular questionnaires and research clinics. Detailed information about ALSPAC is available on the study website (http://www.bristol.ac.uk/alspac), which includes a fully searchable data-dictionary of available data (http://www.bris.ac.uk/alspac/researchers/data-access/data-dictionary). Self-harm was assessed via self-report questionnaire at age 16 years (mean age of respondents 16 years 8 months, standard deviation [SD] approximately 3 months). The postal questionnaire was sent to 9,383 participants of whom 4,855 (51.7%) returned it and 4,810 completed the self-harm items (Kidger et al., 2012). Ethical approval for the study was obtained from the ALSPAC Law and Ethics committee and local research ethics committees (NHS Haydock REC: 10/H1010/70).

#### Linkage

The Health and Social Care Information Centre (HSCIC) linked ALSPAC participants with the NHS Central Register, with a 99% match rate (Boyd et al., 2013); this was done on the basis of NHS ID number, name, date of birth, and postcode using deterministic linkage.

**FIGURE 1:**
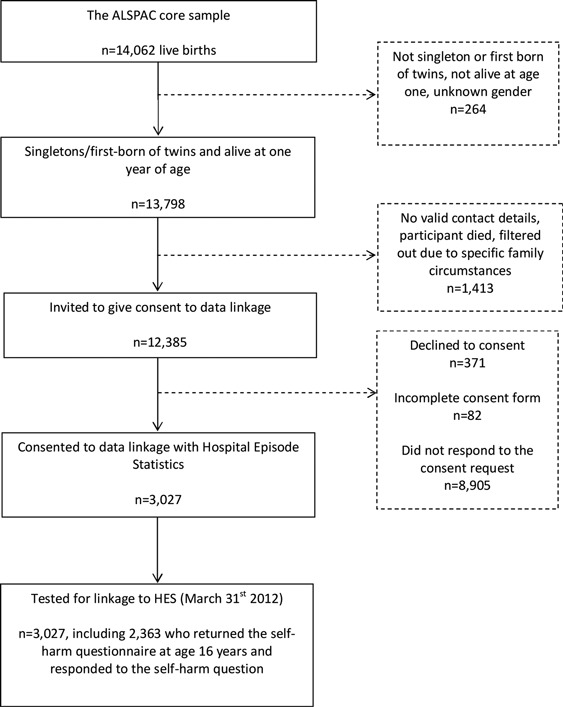
Flow-chart of linkage between the Avon Longitudinal Study of Parents and Children (ALSPAC) birth cohort and the Hospital Episode Statistics database (*HES*).

When the ALSPAC children reached adulthood (age 18), they were invited to enroll in the study in their own right and to consent to the extraction and use of their health records. Through the Project to Enhance ALSPAC through Record Linkage (PEARL) http://www.bristol.ac.uk/alspac/participants/playingyourpart/ information and consent forms were posted to 12,385 of the participants eligible to be included in this investigation (singletons/first born twins from the ALSPAC core enrolled sample who were alive at 1 year. See Figure 1). Of those invited to consent (*n* = 12,385), 3,027 (24.4%) consented to data linkage by the study cut-off date, 8,905 (71.9%) did not respond to the consent request, and 82 (0.7%) returned an incomplete consent form. Only 371 (3.0%) declined to consent.

#### The Hospital Episode Statistics Database (HES)

The HES database (Copyright © 2012, re-used with the permission of The Health and Social Care Information Centre. All rights reserved) contains information about hospital presentations and admissions for all NHS hospitals in England; it contains admissions data from 1989 onwards, outpatient data from 2003 onwards and A&E data from 2007 onwards (http://www.hscic.gov.uk/hes). Of the 3,027 individuals who consented to data linkage (see above) 2,957 individuals (97.7%) had an existing linkage to the NHS central register, which in turn provided a means to identify the individuals’ secondary care records contained in the HES database. The remaining 70 cases were linked to HES using NHS ID number, name, and date of birth. In this scenario “linkage” refers to the process of testing if the ALSPAC participants had any HES records rather than the actual identification and extraction of a record. We make this distinction as some individuals will genuinely not have any HES records, while others may have a HES record which we failed to identify during the linkage process. In March 2013 the NHS Health and Social Care Information Centre (HSCIC) extracted the hospital admissions records of 2,988 participants, although we consider the denominator to be the 3,027 cases tested for linkage.

#### The Clinical Practice Research Datalink (CPRD)

The CPRD is an anonymized database of primary care records of around 5 million (∼8%) patients in the UK. Linkage between ALSPAC and the CPRD was conducted by the NHS Information Centre (NHS IC) as a trusted third party. With approval from the NIGB Ethics and Confidentiality Committee, the NHS IC identified ALSPAC eligible individuals who also appeared in the CPRD, and sent an anonymized linking dataset to be stored securely at the CPRD where the data were merged and analyzed. This particular linkage does not require consent above and beyond the consent obtained for participation in ALSPAC. However, any participants who did not agree to their health records being extracted (via the PEARL consent request described above) were excluded (*n* = 3).

**FIGURE 2:**
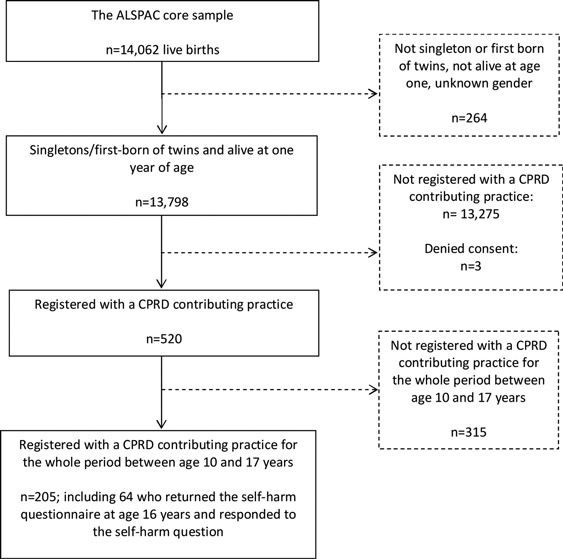
Flow-chart of linkage between the Avon Longitudinal Study of Parents and Children (ALSPAC) birth cohort and the Clinical Practice Research Datalink (CPRD).

Of the live births linked by the NHS IC that appeared in the CPRD, 520 were in the sub-sample eligible for this investigation (singletons/first born twins from the ALSPAC core enrolled sample who were alive at 1 year). The sample was further restricted to individuals who were registered with a CPRD-contributing practice for the entire period between age 10 and 17 years (*n* = 205) (Figure 2), to ensure that there were no breaks in the patients’ records. We did not examine CPRD records before the age of 10 years, as self-harm before this age is rare.

### Measures

History of self-harm was assessed in the ALSPAC cohort, the Hospital Episode Statistics Database (Secondary Care) and the Clinical Practice Research Datalink (Primary Care). The methods of assessment for each data source are described below. Data on psychosocial characteristics were also collected in ALSPAC.

#### The Avon Longitudinal Study of Parents and Children (ALSPAC)

The self-harm questions used in the age 16 self-report questionnaire were based on those used in the CASE study (Madge et al., 2008). Participants who responded positively to the item “have you ever hurt yourself on purpose in any way (e.g., by taking an overdose of pills or by cutting yourself)?” were classified as having a lifetime history of self-harm.

Those who answered “yes” to having self-harmed were then asked further closed response questions, including how long ago they last hurt themselves (in the last week, more than a week ago but in the last year, more than a year ago), the reasons for self-harm the last time they hurt themselves on purpose (six response categories), and whether they had ever seriously wanted to kill themselves when self-harming (Kidger et al., 2012). Participants were classified as having a lifetime history of suicidal self-harm if they selected “I wanted to die” as a reason for harming themselves on the most recent occasion, or if they reported they had ever seriously wanted to kill themselves when self-harming (Mars et al., 2014).

The same question was used to assess lifetime self-harm at age 18 years, using the self-administered computerized version of the Clinical Interview Schedule-Revised (CIS-R) (Lewis, Pelosi, Araya, & Dunn, 1992). There is close agreement between the self-administered computerized version and the interviewer administrated versions of the CIS-R (Bell, Watson, Sharp, Lyons, & Lewis, 2005; Lewis, 1994; Patton et al., 1999).

#### Psychosocial Characteristics

We examined key psychosocial characteristics assessed previously in ALSPAC to identify factors associated with inconsistencies in reporting self-harm over time. The following variables were used: (1) participant's gender, (2) ethnicity, (3) parent social class (professional/managerial or other occupations; the highest of maternal or paternal social class was used), (4) highest maternal educational attainment (less than O-level, O-level, A-level, or university degree) measured during pregnancy (O-levels and A-levels are school qualifications taken around age 16 and 18 years respectively), (5) child IQ assessed using the Wechsler intelligence test for children (WISC-III) (Wechsler, 1991) at age 8 years (6) depression symptoms, assessed at age 16 and 18 years using the short Moods and Feelings Questionnaire (SMFQ), a score of 11 or more on the SMFQ was taken as indicative of depressive symptoms (Patton et al., 2008) and (7) depressive disorder, assessed at age 18 years using the CIS-R.

#### The Hospital Episode Statistics Database (HES)

We used an extract of the HES data including hospital admissions for self-harm (ICD 10 codes Y10–Y34, X60–X84 and X40–X49), A&E attendances for self-harm (A&E diagnostic codes 141/142 “poisoning (inc overdose) due to prescriptive/proprietary drugs,” or reason for A&E attendance coded as “deliberate self-harm”) and hospital admissions for a mental health condition(s) (ICD-10 codes F00–F99). Further details can be found in Appendix 1. While X40–X49 are coded as accidental poisoning, previous studies indicate that they are also used for self-harm. The date of hospital attendance was cross-referenced with the date of questionnaire completion to identify whether events occurred before or after completion of the self-harm questionnaire. Although A&E data is recorded in HES, it is only available from 2007 onwards and is likely to be under-reported. For example, in the extracted data, all but two self-harm hospital admissions were recorded as having come via A&E (the remaining two admissions were emergency referrals by GP); however, two-thirds of hospital admissions had no corresponding A&E record for self-harm. For this reason, we have focused primarily on hospital admissions data in this paper, as this is known to be more complete. The findings for A&E only data are also presented, but need to be interpreted with caution.

#### The Clinical Practice Research Datalink (CPRD)

Cases of self-harm occurring in the CPRD until December 31, 2011 were identified using appropriate Read codes for attempted suicide and self-harm (see Appendix 2) (Thomas et al., 2013).

### Analysis Plan

#### Non-Response

We examined whether there was an association between questionnaire response and medically recorded self-harm by comparing the prevalence of self-harm in HES and the CPRD among those who completed and did not complete the self-harm questionnaire at age 16 years.

#### Agreement Between Self-Report and Medically Recorded Self-Harm Events

We compared self-reported self-harm episodes with events recorded in HES and the CPRD, in order to identify instances in which self-harm was inconsistently reported.

#### Consistency in Self-Report Over Time

We investigated inconsistency in reporting of lifetime self-harm over time between age 16 and 18 years in ALSPAC cohort. Participants who reported no self-harm, or reported self-harm for the first time at age 18 years were excluded from these analyses.

Characteristics associated with inconsistent reporting of self-harm over time were also examined using logistic regression.

## RESULTS

### Self-Harm

#### HES

Of the 3,027 ALSPAC participants tested for linkage with HES (hospital records), 54 (1.8%) had one or more self-harm events recorded in HES, including 41 participants with at least one recorded hospital admission for self-harm, and 18 (0.6%) with at least one recorded “A&E only” attendance for self-harm (i.e., A&E attendance without subsequent hospital admission). It is notable that 66% of individuals who were admitted to hospital following self-harm had no corresponding A&E record for self-harm. Eighty-two (2.7%) had at least one hospital admission for a mental health condition recorded in HES. Of the 3,027 individuals tested for linkage, 2,363 (78.1%) completed the self-harm questionnaire at age 16 years.

#### CPRD

Of the 205 ALSPAC participants registered with a CPRD contributing practice between age 10 and 17 years, 64 (31.2%) completed the self-harm questionnaire at age 16 years. Only 6 participants (2.9%) had a relevant self-harm Read code recorded in the CPRD.

### Non-Response

#### HES

The prevalence of hospital admissions for self-harm and mental health conditions recorded in HES was higher among those who did not complete the self-harm questionnaire at age 16 years than among those who did (Table 2) (self-harm hospital admissions: 2.0% in non-responders vs. 1.2% in responders, difference = 0.8%, 95% CI −0.4–1.9%, *P* = 0.128; mental health hospital admissions 4.8 vs. 2.1%, difference = 2.7%, 95% CI 1.0–4.4%, *P* < 0.001). The same pattern of results was found for A&E only self-harm attendances (1.1 vs. 0.5%, difference =0.6%, 95% CI −0.2–1.4%, *P* = 0.081).

**TABLE 2.** Differences In Prevalence of Hospital Admissions For Self-Harm and Mental Health Conditions in the Hospital Episode Statistics Database Among Those Who Completed vs. Those Who Did Not Complete the Age 16 Year Self-Harm Questionnaire

	Self-harm questionnaire data *n* = 2,363 N (%)	No self-harm questionnaire data *n* = 664 N (%)	Difference (95% CI)	*P* value
Admitted to hospital for self-harm	28 (1.2%)	13 (2.0%)	0.8% (−0.4%, 1.9%)	0.128
Admitted to hospital for mental health problem	50 (2.1%)	32 (4.8%)	2.7% (1.0%, 4.4%)	<0.001

#### CPRD

Two of the 6 individuals with a self-harm Read code recorded in the CPRD completed the age 16 self-harm questionnaire. There was no evidence of a difference in prevalence between questionnaire responders and non-responders (2.8% in non-responders vs. 3.1% in responders, difference = 0.3%, 95% CI −4.8–5.4%, *P* = 0.910). These findings need to be interpreted with caution, given the small number of ALSPAC individuals with a self-harm Read code recorded in the CPRD (*n* = 6).

### Agreement Between Self-Report and Medical Records

#### HES

Of the 2,363 individuals tested for linkage who completed the self-harm questionnaire at age 16 years, 419 (17.7%) reported a history of self-harm. Only 12 (2.9%; 95% CI 1.5–5.9%) of these episodes were recorded in HES.

There were 15 self-harm hospital attendances recorded in HES *prior to completion of the self-harm questionnaire* (12 admissions and 3 A&E only attendances). Three (20%; 95% CI 4–48%) of these episodes were not reported by ALSPAC participants on the questionnaire (1/12 admissions and 2/3 A&E only attendances).

#### CPRD

Both of the self-harm events recorded in the CPRD were reported by participants on the self-harm questionnaire; however, neither participant reported having sought help for self-harm from their GP (a consultation with the GP would be necessary in order for a self-harm Read code to be recorded in the CPRD).

### Consistency of Reporting of Self-Harm Over Time

Five hundred and eighty nine individuals reported lifetime self-harm at age 16 years and provided information on self-harm at age 18 years. Of these, 385 (65.4%) reported self-harm consistently at both time points, and 204 individuals (34.6%) reported self-harm inconsistently, i.e., reported lifetime self-harm at age 16 years but not at age 18 years.

#### Characteristics Associated With Consistency in Reporting of Self-Harm Over Time

Compared with those who reported self-harm consistently over time, those who reported self-harm inconsistently were less likely to have evidence of depression at age 16 and 18 years, were less likely to have self-harmed in the year prior to the age 16 year questionnaire, and were less likely to have harmed with suicidal intent by age 16 years (Table 3). There was little evidence for differences according to gender, social class, IQ, maternal education or ethnicity (Table 3).

**TABLE 3.** Psychosocial Characteristics Associated With Inconsistent Reporting of Self-Harm Episodes Over Time

	Self-harm reported consistently (*n* = 385)	Self-harm reported inconsistently (*n* = 204)	OR [95%CI]	*P* value
Female gender, *n* (%)	312 (81.0%)	162 (79.4%)	0.90 [0.59, 1.38]	0.636
Parental social class, (pregnancy), *n* (%)				
Other	131 (36.1%)	62 (32.5%)		
Professional/managerial	232 (63.9%)	129 (67.5%)	0.85 [0.59, 1.23]	0.395
Mother's education (pregnancy), *n* (%)^*a*^				
<O-level	60 (15.9%)	29 (14.6%)		
O-level	141 (37.4%)	69 (35.2%)		
Degree/A level	176 (46.7%)	100 (50.2%)	0.91 [0.72, 1.16]	0.441
Ethnicity				
White	361 (96.8%)	190 (96.5%)		
Non-white	12 (3.2%)	7 (3.5%)	0.90 [0.34, 2.33]	0.832
IQ, age 8 years, mean (SD)	108.7 (15.6)	110.6 (14.5)	1.01 [1.00, 1.02]	0.166
Depression symptoms: SMFQ score 11+, age 16 years, *n* (%)	167 (44.2%)	62 (30.7%)	0.56 [0.39, 0.80]	0.002
Depression symptoms: SMFQ score 11+, age 18 years, *n* (%)	159 (46.4%)	59 (31.1%)	0.52 [0.36, 0.76]	0.001
Depressive disorder: CIS-R, age 18 years	83 (21.6%)	15 (7.4%)	0.29 [0.16, 0.52]	<0.001
Past year self-harm, age 16 years	237 (61.9%)	99 (49.3%)	0.60 [0.42, 0.84]	0.003
Lifetime self-harm with suicidal intent, age 16 years	154 (40.2%)	42 (20.9%)	0.39 [0.26, 0.58]	<0.001

*Note.* SMFQ: Short Mood and Feelings Questionnaire; CIS-R: Clinical Interview Schedule revised.

^*a*^OR for maternal education assumes a linear trend across the education categories.

## DISCUSSION

### Main Findings

This study is, as far as we are aware, the first to examine whether the prevalence of medically recorded self-harm differs from prevalence determined by questionnaire response in a community-based sample of adolescents. We also investigated the level of agreement between self-reported self-harm history and data obtained from medical records.

We found some evidence for both selective non-participation of individuals with self-harm, and for discrepancies between self-reported and medically recorded self-harm episodes; approximately one-fifth of self-harm events recorded in HES (hospital admissions or A&E presentations) were not reported by participants on the questionnaire. Taken together, these findings suggest that prevalence estimates derived from self-report may underestimate the true rate of adolescent self-harm in the community.

We additionally examined the consistency of self-reported self-harm over time and found that over a third of respondents who reported self-harm at age 16 years said they had never self-harmed when asked at age 18 years. Those who reported self-harm inconsistently over time were less likely to have to have depressive disorder, less likely to have harmed in the year prior to the age 16 year questionnaire and were less likely to have self-harmed with suicidal intent.

### Strengths and Limitations

ALSPAC is a large, population-based study, which is important, given that less than 20% of adolescents who self-harm present to medical services (Hawton et al., 2002; Kidger et al., 2012). We investigated the level of agreement in reports of self-harm both across different sources (self-report and medical records) and over time.

The findings need to be interpreted in light of several limitations. First, we were only able to compare reports among those who had been admitted to hospital or had consulted with their GP. We were also only able to examine self-harm hospital admissions among those who had consented to data linkage (24% of the sub-sample invited to consent) and GP events for those in the CPRD between age 10 and 17 years (1.5% of the sub-sample of 13,798 included in this investigation). These sub samples with available linked records may not be representative of the whole ALSPAC cohort. The issue of required consent has the potential to induce bias in our findings, however using questionnaire data we found little evidence of an association between self-harm and consent to data linkage and so this is unlikely to be a problem in this study. Second, it is likely that cultural differences influence self-reporting of self-harm. The degree of stigma associated with mental illness and self-harm varies around the world (Abdullah & Brown, 2011; Evans-Lacko, Brohan, Mojtabai, & Thornicroft, 2012; Reynders, Kerkhof, Molenberghs, & Van Audenhove, 2014), therefore findings from our study may not be generalizable outside a UK context.

Third, the number of individuals with self-harm recorded in their medical records was small, particularly in the CPRD. This precluded our ability to examine characteristics associated with inconsistent reporting, and limited power to detect differences between questionnaire responders and non-responders. Findings therefore need to be interpreted with caution, and require replication in a larger sample. It is possible that some episodes of self-harm may not have been recorded in the CPRD (Thomas et al., 2013), or may have been missed (i.e., if documented as a free text response rather than a Read code). Fourth, when extracting data from the HES database, we included codes related to accidental poisoning (ICD 10 codes X40–X49) as these codes are often used to indicate self-harm. While some may be true instances of accidental self-poisoning, this is unusual in adolescence.

Finally, self-harm in ALSPAC was assessed via self-report questionnaire at age 16 years and via a self-administered computerized assessment at age 18 years. Although the question used at both time points was identical, the difference in setting may have contributed to the discrepancies in reporting found in this study.

### Comparison With Previous Research

Previous studies investigating inconsistency in reporting of self-harm have typically relied on comparisons between interview and questionnaire responses (Bjärehed et al., 2013; Ougrin & Boege, 2013; Ross & Heath, 2002; Velting et al., 1998). Lower rates of self-harm are usually found when using interview as opposed to questionnaire measures (Evans, Hawton, Rodham, Psychol, & Deeks, 2005). However, the absence of a gold standard assessment for self-harm means that it is not possible to identify which of these measurement approaches is more accurate—the ability to ask additional clarification questions could help to eliminate false positives that arise from inaccurate self-reports (Hawton et al., 2002; Ross & Heath, 2002; Velting et al., 1998), but it is also possible that the loss of anonymity found with interview assessments may result in under-reporting of self-harm (Safer, 1997).

In the Early Developmental stages of Psychopathology Study, Christl et al. (2006) found some evidence for selective non-response as those who reported suicide attempts at baseline were at least 1.6 times more likely to drop out of the study than those without suicidal thoughts or behavior. The use of data linkage allows us to extend this work by objectively comparing the prevalence of self-harm among questionnaire responders and non-responders. There was also some evidence for inconsistency between self-reported and medically recorded self-harm. Possible reasons for discrepancies include concerns over stigma, denial, or problems with recall. Individuals may also suppress painful memories such as self-harm or suicidal ideation, which has been suggested as a possible adaptive defensive mechanism (Goldney, Winefield, Winefield, & Saebel, 2009; Klimes-Dougan, Safer, Ronsaville, Tinsley, & Harris, 2007).

Our finding that a third of adolescents were discrepant in their reporting of lifetime self-harm over time is lower than the proportion found by Hart et al. (2013) (approximately two thirds disrepant 1 year after reporting a self-harm event) but similar to findings of other previous longitudinal research (Eikelenboom et al., 2014, Christl et al., 2006), all of which investigated reporting of suicide attempts. Inconsistent reporting has also been shown for other stigmatized behaviors such as drug use (Percy, McAlister, Higgins, McCrystal, & Thornton, 2005). We extend this research by examining consistency in reporting of self-harm regardless of suicidal intent, and by examining various characteristics associated with discrepant reporting. Similar to Christl et al. (2006) and Eikelenboom et al. (2014), we found greater consistency in reporting among those with psychopathology. We also found individuals were more likely to report self-harm consistently if they had harmed with suicidal intent during their lifetime, and if they had self-harmed in the year prior to questionnaire completion. This could suggest that more severe self-harm episodes and those that are more recent are more likely to be recalled by participants and may be less subject to reinterpretation. However, in their investigation of suicide attempts in adults, Eikelenboom et al. (2014) found no association between consistency in reporting and the recency of self-harm at baseline. It is also possible that individuals with psychopathology and those who have harmed with suicidal intent may be more likely to continue to self-harm as adults. The reasons for discrepant reporting require further investigation and could include denial, errors in recall, or reinterpretation of the self-harm event. Reports may also be influenced by current mood state, for example depressed mood could lead to enhanced recall of negative events, such as self-harm. Unfortunately, it is not possible to determine in this, or other studies, which of the assessments is more accurate (i.e., whether the first reporting of self-harm is a false positive or whether the second reporting is a false negative).

It is also important to note that while selective non-participation of those with self-harm and inconsistent reporting could result in distorted prevalence estimates, this does not necessarily lead to biased estimates of associations between self-harm and exposure variables (Wolke et al., 2009). Further research is planned to investigate this issue in more detail within the ALSPAC cohort.

## CONCLUSION

In our analyses of the ALSPAC cohort, we have shown that self-harm prevalence estimates derived from self-report are affected by non-response and inconsistent reporting, and likely underestimate the true level of adolescent self-harm in the community. Our findings require replication, but suggest benefits of combining self-report self-harm data with data from medical records. To maximize the potential for this approach would require complete coverage of medical records for the sample in question. In practice achieving this may be restricted by governance requirements based on concerns around the protection of privacy with regard to sensitive information in the situation where individuals have not provided explicit consent. Such concerns may be offset by evidence that data-linkage as we describe here can improve the validity of medical research and thus enhance the potential of research to improve the public good.

## DISCLOSURE

The authors report no conflict of interests

## APPENDIX 1

**List of:** ICD-10 Codes Used to Identify Hospital Admissions for Non-fatal Self-harm in the Hospital Episodes Statistics database (HES)

ICD-10 code	Description
X40	Accidental poisoning by and exposure to nonopioid analgesics, antipyretics and antirheumatics
X41	Accidental poisoning by and exposure to antiepileptic, sedative-hypnotic, antiparkinsonism and psychotropic drugs, not elsewhere classified
X42	Accidental poisoning by and exposure to narcotics and psychodysleptics [hallucinogens], not elsewhere classified
X43	Accidental poisoning by and exposure to other drugs acting on the autonomic nervous system
X44	Accidental poisoning by and exposure to other and unspecified drugs, medicaments and biological substances
X45	Accidental poisoning by and exposure to alcohol
X46	Accidental poisoning by and exposure to organic solvents and halogenated hydrocarbons and their vapors
X47	Accidental poisoning by and exposure to other gases and vapors
X48	Accidental poisoning by and exposure to pesticides
X49	Accidental poisoning by and exposure to other and unspecified chemicals and noxious substances
X60	Intentional self-poisoning by and exposure to nonopioid analgesics, antipyretics and antirheumatics
X61	Intentional self-poisoning by and exposure to antiepileptic, sedative-hypnotic, antiparkinsonism and psychotropic drugs, not elsewhere classified
X62	Intentional self-poisoning by and exposure to narcotics and psychodysleptics [hallucinogens], not elsewhere classified
X63	Intentional self-poisoning by and exposure to other drugs acting on the autonomic nervous system
X64	Intentional self-poisoning by and exposure to other and unspecified drugs, medicaments and biological substances
X65	Intentional self-poisoning by and exposure to alcohol
X66	Intentional self-poisoning by and exposure to organic solvents and halogenated hydrocarbons and their vapors
X67	Intentional self-poisoning by and exposure to other gases and vapors
X68	Intentional self-poisoning by and exposure to pesticides
X69	Intentional self-poisoning by and exposure to other and unspecified chemicals and noxious substances
X70	Intentional self-harm by hanging, strangulation and suffocation
X71	Intentional self-harm by drowning and submersion
X72	Intentional self-harm by handgun discharge
X73	Intentional self-harm by rifle, shotgun and larger firearm discharge
X74	Intentional self-harm by other and unspecified firearm discharge
X75	Intentional self-harm by explosive material
X76	Intentional self-harm by smoke, fire and flames
X77	Intentional self-harm by steam, hot vapors and hot objects
X78	Intentional self-harm by sharp object
X79	Intentional self-harm by blunt object
X80	Intentional self-harm by jumping from a high place
ICD-10 code	Description
X81	Intentional self-harm by jumping or lying before moving object
X82	Intentional self-harm by crashing of motor vehicle
X83	Intentional self-harm by other specified means
X84	Intentional self-harm by unspecified means
Y10	Poisoning by and exposure to nonopioid analgesics, antipyretics and antirheumatics, undetermined intent
Y11	Poisoning by and exposure to antiepileptic, sedative-hypnotic, antiparkinsonism and psychotropic drugs, not elsewhere classified, undetermined intent
Y12	Poisoning by and exposure to narcotics and psychodysleptics [hallucinogens], not elsewhere classified, undetermined intent
Y13	Poisoning by and exposure to other drugs acting on the autonomic nervous system, undetermined intent
Y14	Poisoning by and exposure to other and unspecified drugs, medicaments and biological substances, undetermined intent
Y15	Poisoning by and exposure to alcohol, undetermined intent
Y16	Poisoning by and exposure to organic solvents and halogenated hydrocarbons and their vapors, undetermined intent
Y17	Poisoning by and exposure to other gases and vapors, undetermined intent
Y18	Poisoning by and exposure to pesticides, undetermined intent
Y19	Poisoning by and exposure to other and unspecified chemicals and noxious substances, undetermined intent
Y20	Hanging, strangulation and suffocation, undetermined intent
Y21	Drowning and submersion, undetermined intent
Y22	Handgun discharge, undetermined intent
Y23	Rifle, shotgun and larger firearm discharge, undetermined intent
Y24	Other and unspecified firearm discharge, undetermined intent
Y25	Contact with explosive material, undetermined intent
Y26	Exposure to smoke, fire and flames, undetermined intent
Y27	Contact with steam, hot vapors and hot objects, undetermined intent
Y28	Contact with sharp object, undetermined intent
Y29	Contact with blunt object, undetermined intent
Y30	Falling, jumping, or pushed from a high place, undetermined intent
Y31	Falling, lying, or running before or into moving object, undetermined intent
Y32	Crashing of motor vehicle, undetermined intent
Y33	Other specified events, undetermined intent
Y34	Unspecified event, undetermined intent

## APPENDIX 2

**List of:** Read Codes Used to Identify Suicides and Non-fatal Self-harm in the Clinical Practice Research Datalink (CPRD)

Read code	Description
SL..14	Overdose of biological substance
SL..15	Overdose of drug
SLHz.00	Drug and medicament poisoning not otherwise specified
TK..00	Suicide and self-inflicted injury
TK..11	Cause of overdose—deliberate
TK..12	Injury—self-inflicted
TK..13	Poisoning—self-inflicted
TK..14	Suicide and self-harm
TK..15	Attempted suicide
TK..17	Para-suicide
TK0.00	Suicide + self-inflicted poisoning by solid/liquid substances
TK00.00	Suicide + self-inflicted poisoning by analgesic/antipyretic
TK01.00	Suicide + self-inflicted poisoning by barbiturates
TK01000	Suicide and self-inflicted injury by amylobarbitone
TK01100	Suicide and self-inflicted injury by barbitone
TK01400	Suicide and self-inflicted injury by phenobarbitone
TK02.00	Suicide + self-inflicted poisoning by other sedatives/hypnotics
TK03.00	Suicide + self-inflicted poisoning tranquillizer/psychotropic
TK04.00	Suicide + self-inflicted poisoning by other drugs/medicines
TK05.00	Suicide + self-inflicted poisoning by drug or medicine not otherwise specified
TK06.00	Suicide + self-inflicted poisoning by agricultural chemical
TK07.00	Suicide + self-inflicted poisoning by corrosive/caustic substance
TK0z.00	Suicide + self-inflicted poisoning by solid/liquid substance not otherwise specified
TK1.00	Suicide + self-inflicted poisoning by gases in domestic use
TK10.00	Suicide + self-inflicted poisoning by gas via pipeline
TK11.00	Suicide + self-inflicted poisoning by liquefied petrol gas
TK1y.00	Suicide and self-inflicted poisoning by other utility gas
TK1z.00	Suicide + self-inflicted poisoning by domestic gases not otherwise specified
TK2.00	Suicide + self-inflicted poisoning by other gases and vapors
TK20.00	Suicide + self-inflicted poisoning by motor vehicle exhaust gas
TK21.00	Suicide and self-inflicted poisoning by other carbon monoxide
TK2z.00	Suicide + self-inflicted poisoning by gases and vapors not otherwise specified
TK3.00	Suicide + self-inflicted injury by hang/strangulate/suffocate
TK30.00	Suicide and self-inflicted injury by hanging
TK31.00	Suicide + self-inflicted injury by suffocation by plastic bag
TK3y.00	Suicide + self-inflicted injury by other means than hang/strangle/suffocate
TK3z.00	Suicide + self-inflicted injury by hang/strangle/suffocate not otherwise specified
TK4.00	Suicide and self-inflicted injury by drowning
TK5.00	Suicide and self-inflicted injury by firearms and explosives
TK51.00	Suicide and self-inflicted injury by shotgun
TK52.00	Suicide and self-inflicted injury by hunting rifle
TK54.00	Suicide and self-inflicted injury by other firearm
TK5z.00	Suicide and self-inflicted injury by firearms/explosives not otherwise specified
TK6.00	Suicide and self-inflicted injury by cutting and stabbing
TK60.00	Suicide and self-inflicted injury by cutting
TK60100	Self-inflicted lacerations to wrist
TK60111	Slashed wrists self-inflicted
TK61.00	Suicide and self-inflicted injury by stabbing
TK6z.00	Suicide and self-inflicted injury by cutting and stabbing not otherwise specified
TK7.00	Suicide and self-inflicted injury by jumping from high place
TK70.00	Suicide + self-inflicted injury—jump from residential premises
TK71.00	Suicide + self-inflicted injury—jump from other manmade structure
TK72.00	Suicide + self-inflicted injury—jump from natural sites
TK7z.00	Suicide + self-inflicted injury—jump from high place not otherwise specified
TKx.00	Suicide and self-inflicted injury by other means
TKx0.00	Suicide + self-inflicted injury—jump/lie before moving object
TKx0000	Suicide + self-inflicted injury—jumping before moving object
TKx1.00	Suicide and self-inflicted injury by burns or fire
TKx2.00	Suicide and self-inflicted injury by scald
TKx3.00	Suicide and self-inflicted injury by extremes of cold
TKx4.00	Suicide and self-inflicted injury by electrocution
TKx5.00	Suicide and self-inflicted injury by crashing motor vehicle
TKx6.00	Suicide and self-inflicted injury by crashing of aircraft
TKx7.00	Suicide and self-inflicted injury caustic substance, excluding poison
TKxy.00	Suicide and self-inflicted injury by other specified means
TKxz.00	Suicide and self-inflicted injury by other means not otherwise specified
TKy.00	Late effects of self-inflicted injury
TKz.00	Suicide and self-inflicted injury not otherwise specified
U2..00	[X]Intentional self-harm
U2..11	[X]Self-inflicted injury
U2..12	[X]Injury—self-inflicted
U2..13	[X]Suicide
U2..14	[X]Attempted suicide
U2..15	[X]Para-suicide
U20.00	[X]Intentional self-poisoning/exposure to noxious substances
U20.11	[X]Deliberate drug overdose/other poisoning
U200.00	[X]Intentional self-poisoning/exposure to non-opioid analgesic
U200.11	[X]Overdose—paracetamol
U200.12	[X]Overdose—ibuprofen
U200.13	[X]Overdose—aspirin
U200000	[X]Intentional self-poisoning/exposure to non-opioid analgesic at home
U200100	[X]Intentional self-poisoning non-opioid analgesic at residential institution
U200400	[X]Intentional self-poisoning non-opioid analgesic in street/highway
U200500	[X]Intentional self-poisoning non-opioid analgesic trade/service area
U200y00	[X]Intentional self-poisoning non-opioid analgesic other specified place
U200z00	[X]Intentional self-poisoning non-opioid analgesic unspecified place
U201.00	[X]Intentional self-poisoning/exposure to antiepileptic
U201000	[X]Intentional self-poisoning/exposure to antiepileptic at home
U201z00	[X]Intentional self-poisoning antiepileptic unspecified place
U202.00	[X]Intentional self-poisoning/exposure to sedative hypnotic
U202.11	[X]Overdose—sleeping tablets
U202.12	[X]Overdose—diazepam
U202.13	[X]Overdose—temazepam
U202.15	[X]Overdose—nitrazepam
U202.16	[X]Overdose—benzodiazepine
U202.17	[X]Overdose—barbiturate
U202.18	[X]Overdose—amobarbital
U202000	[X]Intentional self-poisoning /exposure to sedative hypnotic at home
U202400	[X]Intentional self-poisoning sedative hypnotic in street/highway
U202y00	[X]Intentional self-poisoning sedative hypnotic other specified place
U202z00	[X]Intentional self-poisoning sedative hypnotic unspecified place
U204.00	[X]Intentional self-poisoning/exposure to psychotropic drug
U204.11	[X]Overdose—antidepressant
U204.12	[X]Overdose—amitriptyline
U204.13	[X]Overdose—SSRI
U204000	[X]Intentional self-poisoning /exposure to psychotropic drug at home
U204100	[X]Intentional self-poisoning psychotropic drug at residential institution
U204y00	[X]Intentional self-poisoning psychotropic drug other specified place
U204z00	[X]Intentional self-poisoning psychotropic drug unspecified place
U205000	[X]Intentional self-poisoning/exposure to narcotic drug at home
U205y00	[X]Intentional self-poisoning narcotic drug other specified place
U205z00	[X]Intentional self-poisoning narcotic drug unspecified place
U206.00	[X]Intentional self-poisoning/exposure to hallucinogen
U206400	[X]Intentional self-poisoning hallucinogen in street/highway
U207.00	[X]Intentional self-poisoning/exposure to other autonomic drug
U207000	[X]Intentional self-poisoning/exposure to other autonomic drug at home
U207z00	[X]Intentional self-poisoning other autonomic drug unspecified place
U208.00	[X]Intentional self-poisoning/exposure to other/unspecified drug/medicament
U208400	[X]Intentional self-poisoning other/unspecified drug/medication in street/highway
U208y00	[X]Intentional self-poisoning other/unspecified drug/medication other specified place
U208z00	[X]Intentional self-poisoning other/unspecified drug/medication unspecified place
U20A.00	[X]Intentional self-poisoning organic solvent, halogen hydrocarbon
U20A.11	[X]Self-poisoning from glue solvent
U20A000	[X]Intentional self-poisoning organic solvent, halogen hydrocarbon, home
U20A400	[X]Intentional self-poisoning organic solvent, halogen hydrocarbon, in highway
U20Az00	[X]Intentional self-poisoning organic solvent, halogen hydrocarbon, unspecified place
U20B.00	[X]Intentional self-poisoning/exposure to other gas/vapor
U20B.11	[X]Self carbon monoxide poisoning
U20B000	[X]Intentional self-poisoning/exposure to other gas/vapor at home
U20B200	[X]Intentional self-poisoning other gas/vapor school/public admin area
U20By00	[X]Intentional self-poisoning other gas/vapor other specified place
U20Bz00	[X]Intentional self-poisoning other gas/vapor unspecified place
U20C.00	[X]Intentional self-poisoning/exposure to pesticide
U20C.11	[X]Self-poisoning with weedkiller
U20C.12	[X]Self-poisoning with paraquat
U20C000	[X]Intentional self-poisoning/exposure to pesticide at home
U20Cy00	[X]Intentional self-poisoning pesticide other specified place
U20y.00	[X]Intentional self-poisoning/exposure to unspecified chemical
U20y000	[X]Intentional self-poisoning/exposure to unspecified chemical at home
U20y200	[X]Intentional self-poisoning unspecified chemical school/public admin area
U20yz00	[X]Intentional self-poisoning unspecified chemical unspecified place
U21.00	[X]Intentional self-harm by hanging/strangulation/suffocation
U210.00	[X]Intentional self-harm by hanging/strangulation/suffocation at home
U211.00	[X]Intentional self-harm by hanging/strangulation/suffocation occurrence at residential institution
U21y.00	[X]Intentional self-harm by hanging/strangulation/suffocation other specified place
U21z.00	[X]Intentional self-harm by hanging/strangulation/suffocation unspecified place
U22.00	[X]Intentional self-harm by drowning and submersion
U221.00	[X]Intentional self-harm by drowning/submersion occurrence at residential institution
U22y.00	[X]Intentional self-harm by drowning/submersion occurrence at other specified place
U22z.00	[X]Intentional self-harm by drowning/submersion occurrence at unspecified place
U24.00	[X]Intentional self-harm by rifle shotgun/larger firearm discharge
U241.00	[X]Intentional self-harm by rifle shotgun/larger firearm discharge occurrence at residential institution
U242.00	[X]Intentional self-harm by rifle shotgun/larger firearm discharge in school/public admin area
U25.00	[X]Intentional self-harm by other/unspecified firearm discharge
U250.00	[X]Intentional self-harm other/unspecified firearm discharge occurrence at home
U26.00	[X]Intentional self-harm by explosive material
U27.00	[X]Intentional self-harm by smoke, fire and flames
U270.00	[X]Intentional self-harm by smoke fire/flames occurrence at home
U274.00	[X]Intentional self-harm by smoke fire/flame occurrence in street/highway
U27z.00	[X]Intentional self-harm by smoke fire/flames occurrence in unspecified place
U28.00	[X]Intentional self-harm by steam hot vapors/hot objects
U280.00	[X]Intentional self-harm by steam hot vapors/hot objects occurrence at home
U28z.00	[X]Intentional self-harm by steam hot vapors/hot objects occurrence in unspecified place
U29.00	[X]Intentional self-harm by sharp object
U290.00	[X]Intentional self-harm by sharp object occurrence at home
U291.00	[X]Intentional self-harm by sharp object occurrence at residential institution
U294.00	[X]Intentional self-harm by sharp object occurrence in street/highway
U29y.00	[X]Intentional self-harm by sharp object occurrence at other specified place
U29z.00	[X]Intentional self-harm by sharp object occurrence at unspecified place
U2A.00	[X]Intentional self-harm by blunt object
U2A0.00	[X]Intentional self -arm by blunt object occurrence at home
U2A1.00	[X]Intentional self -arm by blunt object occurrence at residential institution
U2A3.00	[X]Intentional self -arm by blunt object occurrence at sports/athletic area
U2B.00	[X]Intentional self-harm by jumping from a high place
U2B0.00	[X]Intentional self-harm by jumping from high place occurrence at home
U2B4.00	[X]Intentional self-harm by jumping from high place occurring in street/highway
U2B6.00	[X]Intentional self-harm by jumping from high place industrial/construction area
U2By.00	[X]Intentional self-harm by jumping from high place occurrence other specified place
U2Bz.00	[X]Intentional self-harm by jumping from high place occurrence unspecified place
U2C.00	[X]Intentional self-harm by jumping/lying before moving object
U2C1.00	[X]Intentional self-harm by jumping/lying before moving object occurrence at residential institution
U2C4.00	[X]Intentional self-harm by jumping/lying before moving object occurrence in street/highway
U2Cy.00	[X]Intentional self-harm by jumping/lying before moving object occurrence other specified place
U2D.00	[X]Intentional self-harm by crashing of motor vehicle
U2D0.00	[X]Intentional self-harm by crashing of motor vehicle occurrence at home
U2D4.00	[X]Intentional self-harm by crashing of motor vehicle occurrence in street/highway
U2D6.00	[X]Intentional self-harm by crashing of motor vehicle occurrence industrial/construction area
U2E.00	[X]Self-mutilation
U2y.00	[X]Intentional self-harm by other specified means
U2y0.00	[X]Intentional self-harm by other specified means occurrence at home
U2y1.00	[X]Intentional self-harm by other specified means occurrence at residential institution
U2yz.00	[X]Intentional self-harm by other specified means occurrence at unspecified place
U2z.00	[X]Intentional self-harm by unspecified means
U2z0.00	[X]Intentional self-harm by unspecified means occurrence at home
U2z2.00	[X]Intentional self-harm by unspecified means occurrence school/institution/public administrative area
U2zy.00	[X]Intentional self-harm by unspecified means occurrence other specified place
U2zz.00	[X]Intentional self-harm by unspecified means occurrence at unspecified place
U30.11	[X]Deliberate drug poisoning
U41.00	[X]Hanging strangulation + suffocation undetermined intent
U44.00	[X]Rifle shotgun + larger firearm discharge undetermined intent
U45.00	[X]Other + unspecified firearm discharge undetermined intent
U4B.00	[X]Falling jumping/pushed from high place undetermined intent
U4Bz.00	[X]Fall jump/push from high place undetermined intent occurring at unspecified place
U72.00	[X]Sequelae of intentional self-harm assault + event of undetermined intent
U720.00	[X]Sequelae of intentional self-harm
ZRLfC12	Health of the Nation Outcome Scales item 2—non-accidental self-injury
ZX..00	Self-harm
ZX..11	Self-damage
ZX1.00	Self-injurious behavior
ZX1.12	SIB—self-injurious behavior
ZX1.13	Deliberate self-harm
ZX11.00	Biting self
ZX11.11	Bites self
ZX12.00	Burning self
ZX13.00	Cutting self
ZX13.11	Cuts self
ZX15.00	Drowning self
ZX18.00	Hanging self
ZX19.00	Hitting self
ZX19100	Punching self
ZX19200	Slapping self
ZX1B.00	Jumping from height
ZX1B100	Jumping from building
ZX1B200	Jumping from bridge
ZX1B300	Jumping from cliff
ZX1C.00	Nipping self
ZX1E.00	Pinching self
ZX1G.00	Scratches self
ZX1H.00	Self-asphyxiation
ZX1H100	Self-strangulation
ZX1H200	Self-suffocation
ZX1I.00	Self-scalding
ZX1J.00	Self-electrocution
ZX1K.00	Self-incineration
ZX1K.11	Setting fire to self
ZX1K.12	Setting self alight
ZX1L.00	Self-mutilation
ZX1L100	Self-mutilation of hands
ZX1L200	Self-mutilation of genitalia
ZX1L300	Self-mutilation of penis
ZX1L600	Self-mutilation of ears
ZX1LD00	[X]Self-mutilation
ZX1M.00	Shooting self
ZX1N.00	Stabbing self
ZX1Q.00	Throwing self in front of train
ZX1Q.11	Jumping under train
ZX1R.00	Throwing self in front of vehicle
ZX1S.00	Throwing self onto floor
